# A long non-coding RNA, *afu-254*, is required for the oxidative stress response, cell wall stress response, azole susceptibility, and virulence in *Aspergillus fumigatus*

**DOI:** 10.1128/msphere.00297-26

**Published:** 2026-06-09

**Authors:** Ritu Devkota, Nava Raj Poudyal, Jazmin Reyes Servin, Julianna Lenz, Kelly M. Shepardson, Sourabh Dhingra

**Affiliations:** 1 Department of Biological Sciences, Clemson University 2545, Clemson, South Carolina, USA; 2 Eukaryotic Pathogen Innovation Center, Clemson University 2545, Clemson, South Carolina, USA; 3 Molecular Cell Biology Department, University of California Merced 33244, Merced, California, USA; University of Guelph, Guelph, Ontario, Canada

**Keywords:** lncRNA, azole response, virulence, antimorph

## Abstract

Long non-coding RNAs play an important role in stress response in all forms of life; however, a tight regulation of lncRNAs is required for normal function. Abnormal expression of lncRNA is associated with uncontrolled cell growth in many forms of cancer. Recent studies have highlighted the role of lncRNAs in *Aspergillus fumigatus* in azole stress response and virulence. Here, using a transcriptome data set of *A. fumigatus* response to stress, we identified *afu-254* as an 854 bp lncRNA that plays a role in modulating oxidative stress, fungal sub-MIC azole response to posaconazole and itraconazole, cell wall stress, macrophage phagocytosis and killing *ex vivo*, and virulence in an invertebrate model of *Aspergillus* infection. Importantly, *afu-254* does not produce cross-azole susceptible responses and plays a role in fungal azole responses against posaconazole and itraconazole but not voriconazole. Furthermore, we showed that stoichiometric levels of *afu-254* are important for its function, and ectopic overexpression of *afu-254* in the WT strain leads to an antimorph. This phenotype may stem from a higher-order structure that is denatured with heat, indicating the presence of a non-functional isoform. In conclusion, we characterized a novel lncRNA, *afu-254*, that is important for stress response and virulence in the pathogenic fungus *A. fumigatus*.

**IMPORTANCE:**

Failure of azole treatment for invasive *Aspergillus* infection by both drug-resistant and drug-sensitive isolates is an area of concern and global importance. Fungal stress response is multifaceted, and long non-coding RNAs have emerged as important players in mediating it, including regulating responses to azole antifungals. Here, we have identified a long non-coding RNA, *afu-254*, that plays a role in modulating fungal response to oxidative stress, cell wall stress, azole stress, immune cell stress, and virulence in an invertebrate model of invasive *Aspergillus* infection.

## INTRODUCTION

*Aspergillus fumigatus*, in the genus *Aspergillus*, is the leading cause of invasive pulmonary aspergillosis (IPA) (1). It is ubiquitous in nature and mainly affects people with a compromised immune system. Factors such as prolonged neutropenia, hematologic malignancy, and prolonged use of corticosteroids have all been linked to the disease; however, patient populations with mild or no immunosuppression are increasingly becoming susceptible (2, 3). The azole class of drugs is the primary choice and frontline treatment for management of IPA, which includes voriconazole, posaconazole, itraconazole, and isavuconazole (4). However, the recent rise in azole resistance raised the global alarm, resulting in the WHO identifying drug-resistant *A. fumigatus* as a pathogen of critical concern (5). Azole drugs work by interacting with and blocking the Cyp51 enzyme, a sterol 14α-demethylase enzyme, that is part of ergosterol biosynthesis, the primary sterol in *A. fumigatus* membrane (6). However, binding affinities of azoles to Cyp51 differ on the basis of their side-chains (7), and mutations in the Cyp51 protein sequence, along with duplication in the promoter region, are the primary resistance mechanisms (8). Different mutations within Cyp51 have been found to affect how drug binding occurs. For example, the G54 mutation in Cyp51 confers resistance to itraconazole and posaconazole but not to voriconazole (9, 10). Structural studies have identified differential binding of the short-tail (voriconazole) vs. long-tail (posaconazole and itraconazole) azole drugs to mutated Cyp51 as the reason for the lack of cross-resistance (11). Furthermore, MIC changes for posaconazole and itraconazole correlate owing to their structure (12, 13). Drug binding to the target enzyme is and has been defined as a major contributing factor to drug resistance. However, recently, non-genetic changes, including persistence, tolerance, and hetero-resistance, are also associated with fungal response to azole drugs (14, 15).

Non-genetic mechanisms provide a rapid mode of stress adaptation, including drug response. In pathogenic yeast *Candida albicans*, non-genetic mechanisms are termed para-resistance that are heritable (16). In other fungi, such as *Mucor circinelloides*, RNA mechanisms play a role in drug resistance (17). In *S. pombe*, lncRNAs play a role in response to DNA-damaging agents (18). Additionally, lncRNAs play a role in fungal development and pathogenesis (19–22). In *C. auris*, lncRNA DINOR is a global regulator of stress response and virulence. Importantly, DINOR mutants are more susceptible to oxidative stress and anti-fungal drugs (23). Additional stresses, including acidic, alkaline, and oxidative stress responses, have previously been shown to influence fungal response to azole drugs (24, 25).

However, the roles of non-genetic or epigenetic mechanisms of stress adaptation are not well understood in *A. fumigatus*. To this end, long non-coding RNAs (lncRNAs) are becoming increasingly important in regulating non-genetic stress response mechanisms, including drug responses (26). In *A. fumigatus*, genome-wide discovery led to the identification of 1,089 lncRNAs with a role in regulating azole drug response (27). We have previously shown that an lncRNA, *afu-182*, plays a role in fungal response to azole drugs without a change in its minimum inhibitory concentration (15), where others have also shown a role of ncRNA in drug resistance (27).

Thus, to test the additional roles of lncRNAs in stress response, including oxidative stress response, we analyzed the publicly available data sets of lncRNAs in *A. fumigatus* (28–30) and identified that lncRNA *afu-254* was downregulated under oxidative and iron stress individually, and ~90% downregulated in the presence of both stresses (29). Thus, we hypothesized that *afu-254* is important for fungal response to oxidative stress, iron stress, and azole response.

Here, using rapid amplification of cDNA ends (RACE), we show that *afu-254* is an 854 bp RNA with a 60 bp intron that plays a role in oxidative stress response and differentially regulates azole response to long-tail azoles. In addition, *afu-254* is required for virulence in an invertebrate model of invasive aspergillosis. Thus, here we characterize an lncRNA that regulates fungal azole response and does not provide cross-resistance to azoles. Future research will determine the *afu-254* interactome mediating these phenotypes.

## RESULTS

### *afu-254* is an 854 bp-long non-coding RNA

**Fig 1:**
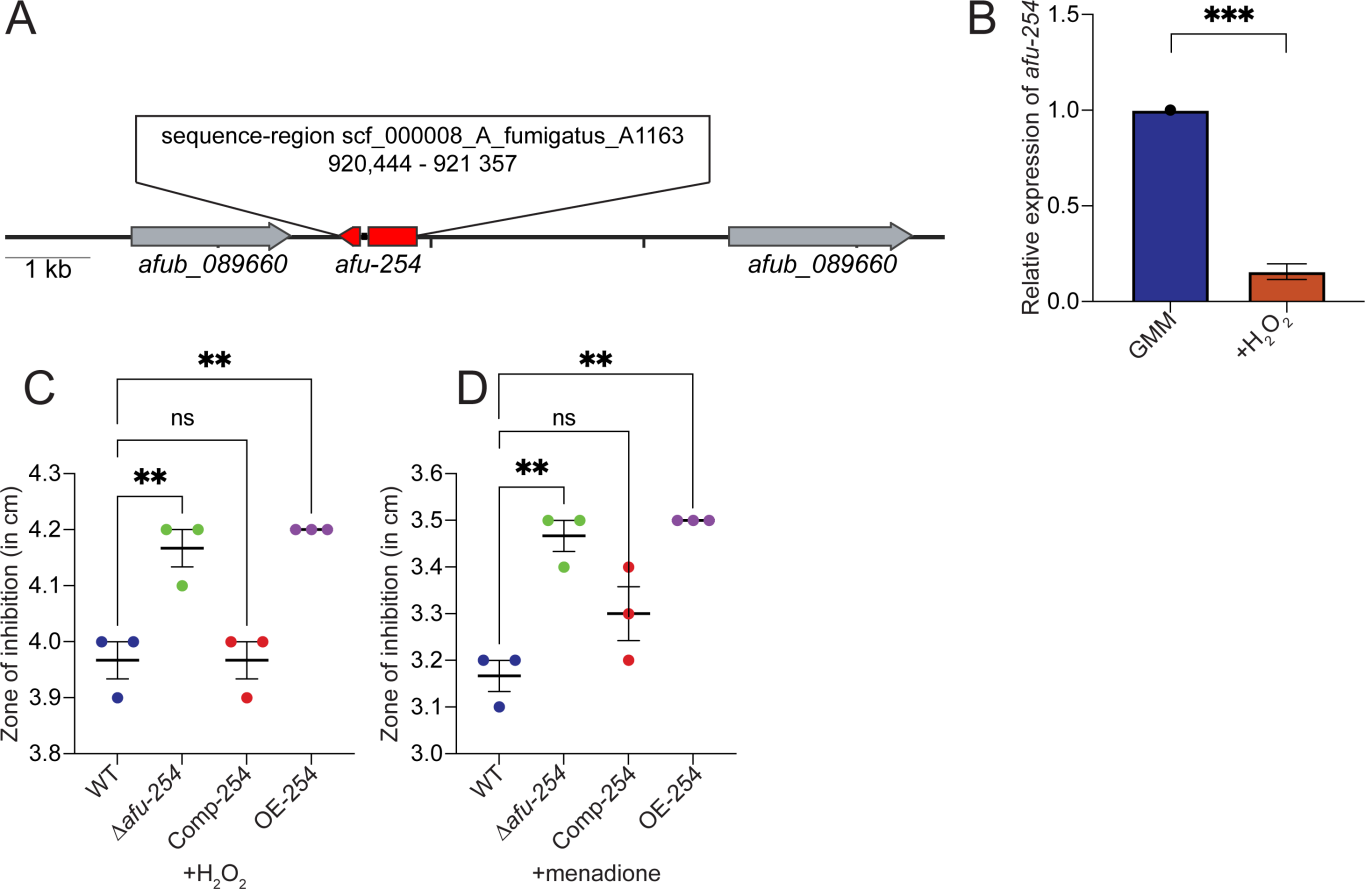
*afu-254* is an lncRNA that regulates fungal oxidative stress response. (**A**) 5′ and 3′ rapid amplification of cDNA ends confirmed the boundary of *afu-254* in the genome as indicated. (**B**) qPCR analysis showing the relative levels of *afu-254* in the presence of oxidative stress compared to GMM. Student’s *t*-test was used to compare the differences in the mean. Fungal strains were inoculated in top agar. After solidification, a core was cut in the center using a sterile pipette tip, and 100 μL of either (**C**) 5 mM H_2_O_2_ or (**D**) 2 mM menadione was added. The zone of inhibition was measured after 36 hours and represented. One-way ANOVA followed by Tukey’s *post hoc* test was used to compare differences in the mean. **P < 0.01, ***P < 0.001, ns—not significant.

To determine the roles of lncRNA in *A. fumigatus* stress response, we identified *afu-254* to be differentially regulated when exposed to oxidative and low iron stress (29). However, *afu-254* is currently annotated as 145 bp ncRNA (Af293 FungiDB accession no. Afu7g04115) (30). To determine the role of *afu-254*, we first identified the boundaries of *afu-254*. We performed 5′ rapid amplification of cDNA ends (RACE) and 3′ RACE (31). We demonstrated that *afu-254* is a 854 bp lncRNA (sequence-region scf_000008_A_fumigatus_A1163 920444-921 357, crick strand) with a 60 bp intron (sequence-region scf_000008_A_fumigatus_A1163 920713-920,772; crick strand) with a Fickett score of 0.39969, a putative ORF of 32AA, a pI of 9.84, and a coding probability of 0.0149372 (32, 33), thus classifying it as a lncRNA (Fig. 1 and Fig. S1). The presence of an intron was confirmed with endpoint PCR (Fig. S1D).

### *afu-254* is differentially regulated by oxidative stress and regulates oxidative stress response in *A. fumigatus*

To confirm the differential expression of *afu-254*, we determined the RNA levels of *afu-254* in GMM and GMM + H_2_O_2_ (3 mM) via quantitative RT-PCR. We observed an 85% reduction (*P* < 0.001) in *afu-254* RNA levels in the presence of oxidative stress (Fig. 1B). To determine the role of *afu-254*, we generated a deletion strain (Δ*afu-254*) and complemented the strain ectopically. We also over-expressed *afu-254* using the *gpdA* promoter from *A. nidulans* as previously described (15). To confirm these strains were correct, we used qPCR to determine the RNA levels of *afu-254* in each strain. We show that there is fourfold over-expression in the OE-254 strain, and no RNA was detected in the Δ*afu-254* strain (Fig. S2). We also performed a northern blot to confirm the presence of RNA in the complement strain (Fig. S2). To determine the role of *afu-254* in regulating fungal response to oxidative stress, we inoculated strains in top agar as previously described (34), punched a hole in the center upon solidification, and added either 5 mM H_2_O_2_ or 2 mM menadione. We incubated the plates for 36 hours and measured the zone of inhibition. Both Δ*afu-254* (*P* < 0.01) and OE-254 (*P* < 0.01) showed increased zone of inhibition compared to WT, indicating *afu-254* is necessary for *A. fumigatus* to ameliorate oxidative stress (Fig. 1C and D). The complement strain reverted growth back to WT. Importantly, the OE-254 strain, like the Δ*afu-254* strain, showed increased susceptibility to oxidative stress. Using a spot assay, by plating *A. fumigatus* conidia in the presence of 3 mM H_2_O_2_, we obtained similar results, further confirming the phenotype (Fig. S2). Thus, *afu-254* plays an important role in regulating fungal oxidative stress response.

### Overexpression of *afu-254* forms a higher-order structure leading to an antimorph

**Fig 2:**
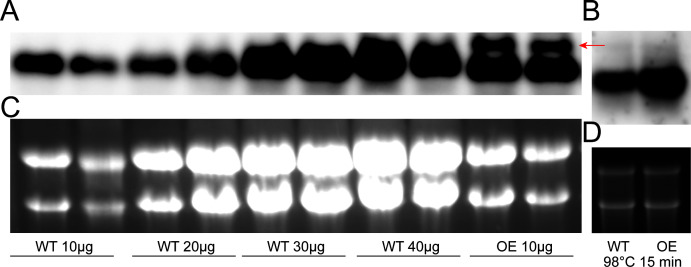
OE-254 constitutes an antimorph. (**A**) WT and OE-254 RNA were loaded in a denaturing formaldehyde gel at the indicated concentration. The samples were heated at 65°C for 5 min before loading and were probed with DIG-labeled *afu-254* sequence. The chemiluminescence image was captured using the Azure C300 imaging system. The OE-254 strain shows the presence of a higher molecular weight band (red arrow). (**B**) The samples were heated at 98°C for 15 min, leading to loss of the higher molecular weight band. (**C and D**). RNA was loaded in a denaturing agarose gel and was imaged before transfer to the nylon membrane. rRNA bands are shown for the quality and quantity of loaded RNA.

Δ*afu-254* strain shows significant growth inhibition in the presence of oxidative stress compared to WT (Fig. 1C and D). Interestingly, the OE-254 strains also showed severe growth attenuation. To confirm the presence of *afu-254* in the OE-254 strain and to determine whether the stoichiometric levels of *afu-254* are important for its functions, we did a northern blot to determine the quantity and quality of *afu-254* transcript. We demonstrate over-expression in the OE-254 mutant; however, we consistently observed the presence of a band above the expected band of OE-254 (Fig. 2A, red arrow). To confirm that the higher-order structure is not just a technical artifact due to the increased amount of target RNA present in the solution, we loaded increasing amounts of WT RNA from 10 to 40 μg (Fig. 2A). We did not see the higher-order structure at any level of loaded RNA, indicating the second higher band is not an artifact. To identify whether this second band is a higher-order structure, we heated the samples at 98°C for 15 min and cooled them immediately before gel loading. As evident in Fig. 2B, we saw complete denaturation of the higher molecular band, and an increase in the weight of the expected monomeric RNA molecule, indicating a possible higher ordered state with *afu-254* acting as an antimorph when over-expressed, resulting in the same oxidative stress phenotype as the deletion mutant.

### *afu-254* regulates posaconazole and itraconazole response but is dispensable for voriconazole response

**Fig 3:**
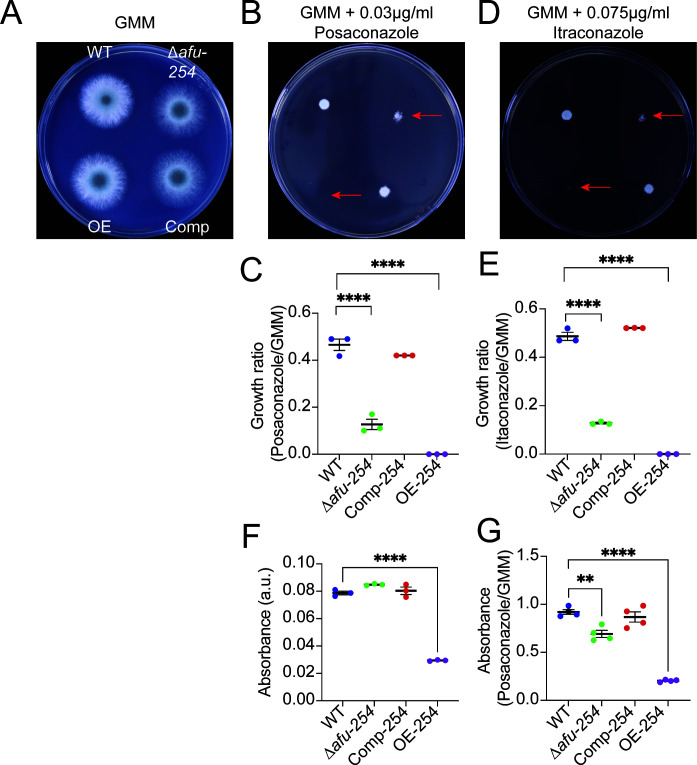
*afu-254* plays a role in fungal response to posaconazole and itraconazole on solid medium and surface-attached cultures. Fungal strains were spot inoculated (10^4^ spores) in (**A**) the absence of drug or the presence of (**B and C**) posaconazole or (**D and E**) itraconazole. Both Δ*afu-254* and OE-254 show increased susceptibility to long-tailed azoles. (**D and E**) Quantitative measurements of fungal growth after 48 hours are represented as a ratio of fungal growth in the presence and absence of the indicated drug. (**F**) *afu-254* plays a role in fungal surface attachment. Fungal strains were allowed to adhere to the abiotic surface for 12 hours. The surface-attached cultures were then (**F**) left untreated (GMM), treated with posaconazole for 8 hours (GMM + 0.2 µg/mL posaconazole), washed, and stained with crystal violet. (**F**) Values represent the absorbance of untreated samples. The OE-254 strain shows decreased surface attachment. (**G**) Values represent the ratio between absorbance measured after azole treatment and fungal attachment before treatment at the 12-hour time point. a.u., arbitrary units. One-way ANOVA followed by Tukey’s *post hoc* test was used to compare differences in the mean. ****P < 0.0001, one-way ANOVA.

We have previously shown that *A. fumigatus* lncRNA regulates fungal response to azole drugs (15). Others have shown that oxidative stress response plays a role in azole response (35, 36). Thus, we inoculated WT, Δ*afu-254*, Comp-254, and OE-254 strains in the presence or absence of posaconazole (0.03 μg/mL), itraconazole (0.075 μg/mL), and voriconazole (0.2 μg/mL). In the presence of posaconazole, the Δ*afu-254* strain is 73% more inhibited compared to WT (Fig. 3B and C, *P* < 0.0001, one-way ANOVA, and Fig. S3A), and the OE-254 strain failed to grow (Fig. 3B and C, *P* < 0.0001, one-way ANOVA, and Fig. S3A). Similarly, we saw a 74% reduction in the Δ*afu-254* strain in the presence of itraconazole (Fig. 3D and E, *P* < 0.0001, one-way ANOVA, and Fig. S3A), and like posaconazole, the OE-254 strain failed to grow in the presence of itraconazole (Fig. 3D and E, *P* < 0.0001, one-way ANOVA, and Fig. S3A). Importantly, no change in voriconazole susceptibility was observed (Fig. S3A). Additionally, the minimum inhibitory concentration of azoles did not change as measured by the broth microdilution method (Fig. S3B). To confirm this is not the growth-stage-specific response, we grew the strains as liquid shaking cultures without or with 0.03 μg/mL posaconazole and weighed the 24-hour dry biomass. Both Δ*afu-2*54 and OE-254 strains failed to produce biomass under liquid shaking conditions (Fig. S3C), indicating the azole susceptibility is not growth condition specific.

### *afu-254* regulates surface attachment and is important for azole response in surface-attached cultures

To determine whether germination is affecting the azole susceptibility phenotypes of *afu-254*, we grew the strains in 24-well plates and allowed the biofilms to develop for 12 hours. After biofilm formation, planktonic cells were washed with PBS, and GMM without or with 0.2 μg/mL of posaconazole was added to the wells for 8 hours. The wells are washed and stained with crystal violet to determine the attached biomass. The OE-254 strain showed a 63% decrease in surface attachment after 20 hours in the absence of azoles (Fig. 3F, *P* < 0.0001). Upon treatment with posaconazole, we normalized the biofilms to untreated controls and observed a 25% reduction in Δ*afu-254* biofilms (Fig. 3G, *P* < 0.01, one-way ANOVA) and 78% reduction in OE-254 biofilms (Fig. 3G, *P* <0.0001, one-way ANOVA) compared to WT. Thus, *afu-254* plays a role in surface attachment and azole response of surface-attached cultures.

### *afu-254* regulates cell wall stress response

**Fig 4:**
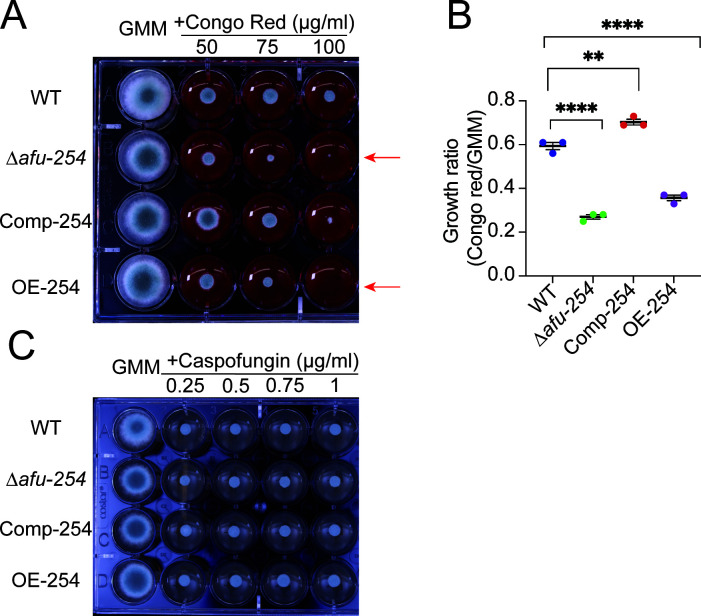
*afu-254* mediates fungal cell wall response to Congo red but not to Caspofungin. Fungal strains were spot inoculated (10^4^ spores) in a 24-well plate in the absence or presence of indicated concentrations of (**A**) Congo red or (**C**) Caspofungin. Both Δ*afu-254* and OE-254 strains are susceptible to Congo red (red arrows) but not to caspofungin. (**B**) Quantitative analysis of fungal growth is represented as a ratio between growth in the presence of CR and the absence of CR. One-way ANOVA followed by Tukey’s *post hoc* test was used to compare differences in the mean. **P < 0.01, ****P < 0.0001.

Hydrogen peroxide-mediated oxidative stress pathway leads to changes in the cell wall integrity pathway (37). Thus, to determine whether *afu-254* plays a role in fungal response to cell wall stress, we incubated the strains in the absence and presence of Congo red, an anionic azo dye that affects cell wall rigidity (38). Δ*afu-254* showed a 54% reduction in growth ratio (Fig. 4A and B, *P* < 0.0001, one-way ANOVA), whereas the OE-254 strain showed a 40% reduction in growth ratio (Fig. 4A and B, *P*<0.0001, one-way ANOVA) compared to WT strains. We did not see a difference when strains were incubated with the echinocandin drug, Caspofungin, indicating a more regulated cell wall stress response (Fig. 4C).

### OE *afu-254* regulates macrophage uptake and killing *ex vivo*

**Fig 5:**
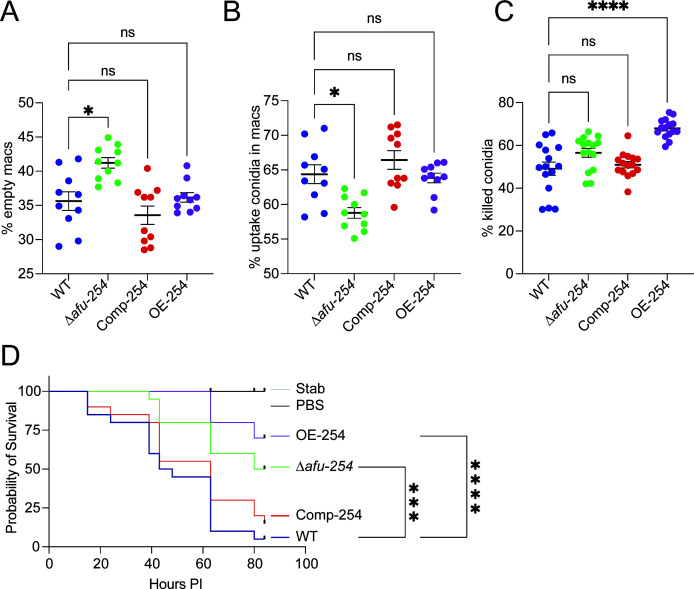
*afu-254* regulates fungal *ex vivo* killing and virulence in an invertebrate model of infection. BMDMs were incubated with conidia from the indicated strain (5:1 conidia to cell) that were biotinylated and stained with AF633 for 3 hours. Cells were collected, and the percentage of live BMDMs (**A**) with (uptake) or (**B**) without (empty) conidia was determined by gating on AF633 + vs AF633- BMDMs using flow cytometry. The Δ*afu-254* strain showed less uptake by BMDMs. The points represent combined data from two experiments. (**C**) BMDMs were incubated with conidia from the indicated strains (5:1 conidia to cell) for 5 hours. Cells were lysed, and CFUs were determined by serial dilution and plating. Results depict the percentage of killed conidia from three independent experiments compared to the starting inoculum. The OE-254 strain shows statistically increased killing by BMDMs, whereas the Δ*afu-254* strain trends toward increased killing, albeit not statistically significant. The Kruskal-Wallis test with Dunn’s multiple comparisons was performed for statistical analyses. All error bars represent standard error. *P < 0.05, ****P < 0.0001. (**D**) *afu-254* plays a role in virulence. *Galleria mellonella* larvae were infected with the 5 × 10^4^ spores of the indicated strains of *A. fumigatus* and monitored. The larvae exhibiting a lack of movement upon physical stimuli were marked as dead (*n* = 20 for all infected larvae, 10 for controls). Log-rank test was used to determine survival distribution, and survival was significantly different for the Δ*afu-254* strain and the OE-254 strain compared to WT (*P* < 0.001 and *P* < 0.0001, respectively).

Reactive oxygen species are detrimental to *A. fumigatus* conidia and are a major weapon used by innate immune cells, including macrophages, to kill *A. fumigatus* conidia (39, 40). As *afu-254* plays a role in oxidative stress response, we tested the ability of immortalized bone-marrow-derived macrophages (BMDMs) to engulf and/or kill conidia using an *ex vivo* adapted FLARE assay and CFU plating assay, respectively (41). For uptake and killing, we incubated conidia from each strain with BMDMs (MOI 5:1) for 3 or 5 hours, respectively. Interestingly, we observe a decrease in uptake of Δ*afu-254* conidia compared to WT but not OE-254 conidia (Fig. 4B, *P* < 0.05, Kruskal-Wallis test). We also observe a significant increase in OE-254 conidia killing (Fig. 5C, *P* < 0.0001, Kruskal-Wallis test). The *ex vivo* killing of Δ*afu-254* trends higher than WT, but is not statistically significant (Fig. 5C, *P* = 0.3616, Kruskal-Wallis test).

### *afu-254* regulates fungal virulence in the *Galleria mellonella* model of infection

The ability to deal with oxidative stress and differences in cell wall stress response has been associated with virulence (1, 42). Thus, we determined the virulence of *A. fumigatus* strains in the *G. mellonella* infection model (43). Worms infected with Δ*afu-254* had a median survival time of 82 hours compared to 45.5 hours for WT (Fig. 5D, *P* < 0.0001, log-rank test). Fifty percent of larvae survived in the Δ*afu-254* group compared to 5% survival in the WT group (*P* < 0.001, Fig. 5D). On the other hand, 70% of larvae infected with the OE-254 strain survived compared to WT (Fig. 5D, median survival time—NA, *P*<0.0001, log-rank test). The experiment was repeated three times with similar results. Thus, *afu-254* plays a role in fungal virulence in *G. mellonella* model of infection.

## DISCUSSION

Long non-coding RNAs play an important role in stress response in all forms of life, and recent findings have shown their importance in azole response in *A. fumigatus* (15, 27). However, whether lncRNAs play a role in modulating other stresses is not entirely known. To understand the role of lncRNAs in pathogenesis-related stress response, we analyzed a previously published transcriptome data set in the absence or presence of oxidative stress, low-iron stress, and a combination of both stresses (29). Based on the previous transcriptomics results and our qPCR data, we determined that non-coding RNA *afu-254* is highly expressed RNA and shows an 85% reduction in the presence of oxidative stress mediated by H_2_O_2_ (Fig. 1B). However, *afu-254* was predicted to be a 171 bp ncRNA, failing to meet the threshold of 200 bp to be classified as lncRNA (28, 30). Previously, the ncRNAs in *A. fumigatus* were determined by a cDNA approach, and more recently, during the course of this study, a genome-wide characterization of lncRNAs in *A. fumigatus* was reported (27, 28). Importantly, *afu-254* was not characterized as an lncRNA in either of those studies; however, longer RNA transcripts are predicted at the locus (30). We did a 5′ and 3′ RACE analysis and characterized *afu-254* as an 854 bp long ncRNA, and the DNA sequence coding for this ncRNA contains a 60 bp intron in the DNA sequence (Fig. 1A and Fig. S1). Under the conditions tested, we only found one transcript; however, whether splicing variants exist is currently under investigation. Also, 5′ RACE is dependent on the ability of reverse transcriptase to amplify full-length cDNA(44), and it is plausible that there is premature elongation termination. However, complementing *afu-254* fragment ectopically reverts the oxidative stress phenotype, cell wall stress phenotype, and virulence back to the WT levels (Fig. 3, Fig. 4 and Fig. 5), indicating functionality. Using species-neutral computational models, the *afu-254* transcript is not predicted to be a protein-coding sequence (32) and thus is classified as an lncRNA.

As our identification and confirmation showed *afu-254* to be differentially regulated by oxidative stress, we grew the strains in the presence and absence of H_2_O_2_ and menadione, which produce peroxide and superoxide, respectively. Both Δ*afu-254* and OE-254 strains showed a significant reduction compared to WT (Fig. 1C and D). Various instances in *A. fumigatus* biology have shown the stoichiometric levels of proteins to be important for their biological function (45); however, this is the first instance where WT levels of an lncRNA are important for its function in *A. fumigatus*. To determine whether the OE-254 strain has a stable transcript, we did northern blot analysis and observed the intact band at the expected size. In addition to the expected band, we also saw a slow-moving band indicating either an *in vitro* artifact, a multimeric complex, or a higher-order structure that is partially denatured (Fig. 2A, red arrow). LncRNAs assemble into three-dimensional secondary structures like stem loops and cloverleaf, and structural conservation is important for their functions (46–48). Secondary structures can coalesce to form higher-order structures like pseudoknots, triplexes, and G-quadraplexes (49, 50). lncRNA can also interact with other RNAs, DNAs, and proteins to bind to chromatin to exert its function (51). To determine whether this is in fact a higher-ordered structure, we boiled the RNA at 98°C for 15 min, followed by rapid cooling on an ice bath before gel loading. We saw an increase in the expected RNA band and a discernible loss of the slow-moving band, indicating loss of higher-ordered structure (Fig. 2B); however, the possibility that it is a DNA/RNA or RNA/RNA hybrid cannot be excluded. Overexpression of lncRNA has long been associated with cancer development and metastasis (52, 53). lncRNAs can act as sponges and decoys that can sequester other regulatory partners from exerting their functions (54). For example, overexpression of lncRNA Xist serves as a competing endogenous RNA (ceRNA) for miR-34a through sponging, leading to overexpression of the MET oncogene that is involved in various cancers (55). In *A. fumigatus*, overexpression of *afu-254* ectopically acts as an antimorph presumably through the formation of a non-functional multimeric form or tertiary structure (56). It is not clear whether this effect is due to *afu-254* driven by a constitutively active *gpdA* promoter or overexpression in general. Further studies will focus on inducible promoters to fine-tune the *afu-254* levels for structural and functional analysis.

Oxidative stress plays a role in fungal response to azole drugs (29). Previous studies on lncRNAs have shown their roles in azole response (15, 27). Thus, we grew the strains in the presence of voriconazole (short-tailed azole), posaconazole, and itraconazole (long-tailed azoles) (11). Surprisingly, we did not see a growth difference in response to voriconazole (Fig. S3A), but both Δ*afu-254* and OE-254 strains were hypersusceptible to both posaconazole and itraconazole (Fig. 3 and Fig. S3). We have previously shown that lncRNA *afu-182* regulates pan-azole response (15); however, unlike *afu-182*, *afu-254*’s response is limited to long-tailed azoles only. Previous studies have shown a strong correlation between posaconazole and itraconazole susceptibility attributed to their structural similarities (12). Azole cross-resistance in *A. fumigatus* is determined by specific mutations in the Cyp51 sequence. For example, G54 and P216 mutations in Cyp51 result in resistance to posaconazole and itraconazole but not to voriconazole (10, 57). The G448S mutation provides a high level of resistance to voriconazole, but a low level of resistance to posaconazole and itraconazole (58), and amino acid variations at M220 lead to pan-azole resistance (59). Promoter duplication, along with amino-acid substitutions, is also a major driver of pan-azole resistance (8). Whether this effect is mediated by a direct role in drug-*Cyp51* interaction or by modulating *cyp51* gene expression is an area of further research. However, we show that both deletion and overexpression of *afu-254* (Δ*afu-254* and OE-254 strains) play a role in fungal response to azole drugs. In addition to DNA changes, drug efflux also plays a role in azole resistance, and voriconazole is a known inducer of MDR (multidrug-resistant pumps) (60). However, unlike voriconazole, long-chain azoles, owing to their long side chain, associate with the membranes more efficiently (61, 62). Thus, posaconazole is a poor substrate for MDR pumps (63). It is plausible that *afu-254* regulates MDR efflux pumps, thus making fungal cells more susceptible to posaconazole and itraconazole but not voriconazole, owing to intracellular concentrations. Future research will determine the intracellular azole concentrations to ascertain the differential mechanism of action against various azoles.

Previous reports have shown that azole treatment leads to increased ROS production, and ameliorating ROS via mutations or upregulation of stress response pathways may play a role in azole resistance (25). We showed that *afu-254* is downregulated under oxidative stress (Fig. 1) but not under azole stress (Fig. S3D); it is also possible that *afu-254* affects mitochondrial dynamics. As long-tailed azoles associate with membranes more efficiently, the observed effect of azoles may partially be due to oxidative stress phenotypes.

We also determined the role of *afu-254* in modulating cell-wall stress response. We plated the strains in the presence of Congo red, an anionic azo dye that binds to chitin and blocks the interaction between chitin and β-1,3 glucans, thereby weakening the cell wall (38). We saw both Δ*afu-254* and OE-254 strains showing increased susceptibility to Congo red (Fig. 4A); however, no difference was observed in response to the Echinocandin drug Caspofungin that targets 1-3-β-D-glucan synthase enzyme, *fksA* (64, 65). It is possible that *afu-254* modulates the levels of cell wall macromolecules or only affects chitin and not β-1,3-glucans. However, the cell wall is the primary interaction site for *A. fumigatus* with immune cells, which function via oxidative and non-oxidative mechanisms for fungal conidia killing. As *afu-254* plays a role in mediating oxidative stress response, we performed an *ex vivo* uptake and killing assay using BMDMs. Our data show decreased uptake but efficient killing of Δ*afu-254* conidia, and increased killing of OE-254 conidia by BMDMs (Fig. 5). Even though we do see a similar trend in phenotypes in Δ*afu-254* and OE-254 strains, it is possible that, due to the higher-order structure of the OE-254 strains, the antimorph effect is beyond the phenotypes detected here, and we are currently parsing out these responses. It is possible that an antimorphic allele in the OE-254 strain affects genomic loci with similar functions, resulting in a hypersusceptible phenotype compared to the null mutant (66). As we only observed cell wall stress phenotype in the presence of chitin-interacting Congo red but not Caspofungin, it is possible that cell wall composition is slightly different in Δ*afu-254* and OE-254 strains. Cell wall beta-glucan becomes exposed in swollen and germinating spores and is recognized by Dectin-1 on resident alveolar macrophages (67). This interaction leads to pro-inflammatory cytokine release (68), whereas chitin recognition is immunomodulatory (69). Chitin can shift the immune response from a Th1 to Th2 and can suppress the activity of IL-1 via increased secretion of IL-1Ra (69, 70). In Δ*afu-254* and OE-254, it is possible that different levels of chitin and β-glucans are exposed, leading to differential immune response.

RNA-mediated mechanisms play a role in fungal pathogenesis in plants, insects, and mammals using varying mechanisms (71, 72). Thus, to further understand whether the *ex vivo* immune response plays a role in virulence, we used the *Galleria mellonella* model of infection, which has been used as a heterologous host to study host-pathogen interaction (43). Here, we infected the larvae with the WT, Δ*afu-254*, Comp*-*254, and OE-254 strains of *A. fumigatus*. We observed significantly reduced mortality in larvae infected with Δ*afu-254* and OE-254 strains that correlate with the *ex vivo* data (Fig. 5D). It is important to note that catalase-deficient mutants and superoxide dismutase mutants that detoxify peroxides and superoxide, respectively, do not show virulence defects in animal models (73, 74), whereas oxidation resistance protein 1 (OxrA) leads to virulence attenuation. It is well established that *A. fumigatus* has a diverse set of mechanisms to detoxify ROS; however, what genes, if any, are regulated by *afu-254* is not clear. In the future, we plan to study the whole transcriptome response in Δ*afu-254* and OE-254 strains compared to the WT strain.

In summary, we identified that *afu-254* is a long non-coding RNA that plays a role in fungal response to oxidative stress, cell wall stress, and virulence. Additionally, *afu-254* modulates fungal azole response to posaconazole and itraconazole but not voriconazole, both in surface and attached cultures. The exact mechanisms of this lack of cross-resistance phenomenon are not clear, and further investigations, both technical and mechanistic, are required to study and understand the roles of lncRNAs in regulating fungal azole response.

In conclusion, we have shown that non-genetic stress response mechanisms play an important role in *A. fumigatus* biology, pathogenesis, and regulate azole stress response. A detailed understanding of their function is needed to better understand the multifaceted azole drug response toward achieving improved treatment outcomes.

## MATERIALS AND METHODS

### Rapid amplification of cDNA ends

5′ rapid amplification of cDNA end (RACE) was done using 5′ RACE system for rapid amplification of cDNA ends from Invitrogen. Briefly, total RNA was isolated from the CEA10 strain as previously described (75). Briefly, *A .fumigatus* strains (10^6^ spores/mL) were grown in 50 mL of glucose minimal media (GMM) (76) for 24 hours at 37°C with constant shaking at 250 rpm. For total RNA isolation, mycelia were collected via filtration and rapidly frozen in liquid nitrogen. Frozen samples were pulverized in acid guanidinium thiocyanate phenol chloroform (AGPC) solution with 2.3 mm silica-zirconia beads in a bead-beater. Samples were centrifuged to remove the insoluble material. A total of 200 μL of chloroform was added per mL of AGPC (75) and centrifuged. We collected the aqueous phase and added an equal volume of 70% ethanol, and transferred the mixture in a silica column (Epoch Life Sciences) to bind RNA to the silica membrane via centrifugation at 12,000 × *g*. Columns were washed with 70% ethanol twice and were completely dried. RNA was eluted in 50 μL of nuclease-free water. RNA quality was assessed via gel electrophoresis and was quantified with Qantas Fluorometer (Promega, USA) per the manufacturer’s recommendations.

Gene-specific primer SD284 was used for first-strand synthesis. cDNA was tailed with dCTP and was amplified with UAP (Invitrogen, Thermo Fisher) and SD366.

3′ RACE was done as previously described (31). Briefly, first-strand cDNA was synthesized using the Superscript II enzyme with primer Qt. *afu-254* was amplified with primers Q_0_ and SD78.

The resulting fragments were ligated into the pJET1.2 vector (Thermo Fisher) and were sequenced using pJET sequencing primers per the manufacturer’s protocol (Thermo Fisher, USA).

### Strain generation

All strains and primers used in this study are listed in Table S1 and Table S2, respectively. CEA17, a uridine/uracil auxotrophic derivative of CEA10, was used as the host strain for creating the Δ*afu-254* strain used in this study (77). Briefly, a 1 kb region upstream and downstream of the predicted *afu-254* sequence was amplified using primers SD266 and SD267 and SD218 and SD219. A fragment encoding the 5′-orotodine decarboxylase gene from *A. parasiticus* was amplified from pSD38.1 using primers SD1 and SD2. The three resulting fragments were fused via PCR using primers SD222 and SD437.

To obtain the complement *afu-254* plasmid*,* the DNA fragment corresponding to the *afu-254* transcript was amplified with primers SD580 and SD581 that harbor AscI and Not1 restriction sites, respectively. The resulting PCR fragment was digested with AscI and NotI and was ligated into plasmid BS311 harboring the hygromycin resistance gene that was previously digested with AscI and NotI (15).

To generate an over-expression strain, *A. nidulans gpdA* promoter just before the TSS was amplified using primers SD134 and SD103 using *A. nidulans* gDNA as the template (78). The DNA sequence corresponding to the *afu-254* transcript was amplified using SD 468 and SD202 with *A. fumigatus* gDNA as the template. These fragments were fused together via fusion PCR using primers SD136 and SD323 that harbor AscI and NotI sites. The resulting fragment was ligated into plasmid pSD18.1, which contains the *pyrG* gene as a fungal selection marker, previously digested with AscI and NotI.

DNA was transformed into the protoplast using polyethylene glycol mediated transformation as previously described (79).

### DNA isolation

DNA was isolated as described previously. Briefly, *A. fumigatus* spores were inoculated into liquid YGT (0.5% yeast extract, 2% glucose, 1× Hunter’s trace elements [80]) and incubated overnight as stationary cultures. Fungal mycelium was harvested and lyophilized. Dry mycelium was pulverized with 2.3 mm silica beads in a bead beater. One milliliter of LETS (0.1 M LiCl, 10 mM EDTA, 10 mM Tris, pH 8.0, and 0.5% SDS) buffer was used. The samples were centrifuged, and 600 μL of supernatant was mixed with an equal volume of phenol:chloroform:isoamyl alcohol (25:24:1). The mixture was centrifuged at 12,000 × *g*. Aqueous phase was collected, and DNA was pelleted at 4°C with 2.5× volume of 100% ethanol. The DNA pellet was washed with 70% ethanol, dried, and resuspended in 50 μL of nuclease-free water. DNA quality was assessed with gel electrophoresis.

### Southern blot

Southern blot was done using standard techniques (81). Briefly, DNA was digested with EcoRV, run on a 1% agarose gel, and transferred to a positively charged Nylon membrane (Roche, USA) and UV-crosslinked. Membrane was pre-hybridized in EasyHyb, probed with DIG-labeled probe, and was detected using anti-DIG antibody per the manufacturer’s recommendations (Roche). CDP-Star (Roche) was used as a substrate, and a chemiluminescent image was acquired with the Azure C300 imaging system.

### Quantitative RT PCR

RNA was isolated as described above (75). RNA was quantified using a NanoDrop and Quantus fluorometer (Promega). RNA quality was assessed on 1% TAE-agarose gel. Five micrograms of total RNA was treated with RNase-free DNase I (Thermo Fisher) according to the manufacturer’s instructions.

For RT-qPCR, 1 μg of RNA was used for first-strand synthesis using MMLV-reverse transcriptase (Promega) per the manufacturer’s instructions. Second-strand synthesis and quantitative real-time PCR were performed using iTaq Universal SYBR mix (Bio-Rad) in the CFX Connect Real-Time System (Bio-Rad). *afu-254* transcript levels were quantified using primers SD 324 and SD 325. Histone H4 was used for normalization, and transcript levels were quantified using primers SD 375 and SD 376. Student’s *t*-test was used to compare the difference in the mean.

### Endpoint PCR using cDNA template

RNA was isolated and treated with DNAse as described above. One microgram of RNA was converted into cDNA using SuperScript IV reverse transcriptase. Primers SD 201 and SD 78 were used for endpoint PCR. CEA10 gDNA was used as the control.

### Northern analysis

For northern analysis, RNA was isolated as above. Indicated amounts of RNA were loaded onto 1.2% agarose–2.2 M formaldehyde gel and blotted on a positively charged nylon membrane. A DIG-labeled probe was synthesized with primers SD201 and SD188 using the PCR DIG probe synthesis kit per the manufacturer’s recommendations. The membrane was incubated with the probe overnight at 42°C in DIG EasyHyb (Roche). The probe was washed and incubated with DIG antibody per the manufacturer’s recommendation (Roche). The membrane was incubated with ready-to-use CDP STAR substrate (Roche), and chemiluminescence was visualized using the Azure C300 chemiluminescent imaging system.

### Stress test

To test the role of *afu-254* in oxidative stress, cell membrane stress, and cell wall stress response in *A. fumigatus*, serial spore dilutions of indicated strains were prepared and inoculated on the plate containing indicated stresses, along with no-stress control, for 48 hours at 37°C, 5% CO_2_. Colony diameter was measured, and statistical analysis was performed using GraphPad Prism.

Punch hole assay was performed as previously described (45). Briefly, 10^5^ spores per mL in 5 mL of top agar were plated on GMM plates. After solidification, a filter pipette tip (200 μL) was used to core out the center of the plate. A total of 100 μL of either 5% H_2_O_2_ or 2 mM menadione was added, and the zone of inhibition was measured.

All experiments were performed in triplicate and were repeated three times. One-way ANOVA was used to compare sample means, followed by Tukey’s *post hoc* analysis.

### Broth microdilution assay to determine minimum inhibitory concentration

Spore suspensions were adjusted to 5 × 10^5^ spores per mL, and 100 μL of spores was added to 100 μL of drug in GMM in a 96-well plate (16 μg/mL–0.0156 μg/mL). The first well showing no fungal growth was recorded as MIC.

### Biofilm assay

In a 24-well plate, 1 mL of GMM containing 10^5^ spores of *A. fumigatus* strains was added. The plates were centrifuged at 250 × *g* for 10 minutes and incubated at 37°C for 12 hours. The culture supernatant was removed, and wells were washed twice with PBS and stained with 2 mL of 0.1% crystal violet for 10 min. GMM without or with 0.2 μg/mL of posaconazole was added for 8 hours. Wells were then washed twice using autoclaved water to remove excess crystal violet and treated with 1.5 mL of 100% ethanol for 10 minutes. Seventy-five microliters of suspension from each well was transferred to the three wells of a 96-well plate, and absorbance at 600 nm was read using a MultiSkan Sky High Microplate Spectrophotometer. To calculate biofilm formation, the background reading was subtracted from the test reading. For azole-treated samples, the absorbance was normalized to untreated controls. One-way ANOVA was used to compare sample means, followed by Tukey’s *post hoc* analysis.

### *Ex vivo* killing assay

Immortalized C57BL/6 BMDMs (BEI resources; NR-9546) were used for the macrophage killing assay. Briefly, BMDMs were plated at 0.5 × 10^6^ cells/well in a 12-well plate. The next day, conidia from the indicated strains, at a ratio of 5:1 (conidia:cells), were added to the BMDMs, centrifuged at 1,000 rpm for 2 minutes, and then incubated for 5 hours at 37°C, 5% CO_2_. Following incubation, cells were lysed for 2 minutes using ice-cold water. Serial dilutions of the wells were made in PBS and plated on GMM plates. Plates were incubated at 37°C for 48 hours, and CFUs were counted.

### Macrophage uptake assay

BMDMs were plated at 0.5 × 10^6^ cells/well in a 12-well plate. The next day, the conidia biotinylation protocol from (41) was used to label each strain with AF633. Briefly, conidia from each of the four strains were rotated in Biotin-XX, SSE at a final concentration of 5 μg/mL in 50 mM carbonate buffer for 1 hour. Following incubation, unbound biotin was neutralized using 0.1 M Tris-HCl and rotated at room temperature for 15 minutes. Conidia were then resuspended in PBS with 0.02 mg/mL of AF633, incubated for 45 minutes with constant rotation, and protected from light until experimental use. Following labeling, each of the five biotinylated strains was added to the corresponding wells at a ratio of 5:1 (conidia: cell) and incubated for 3 hours at 37°C, 5% CO_2_. After incubation, wells were washed with PBS and suspended in fluorescence-activated cell sorting (FACS) buffer (1% fetal bovine serum-PBS). Wells were gently scraped with a cell scraper to resuspend BMDMs. Cells were stained with DAPI for L/D detection and were analyzed using the BD LSR II flow cytometer. Gating on BMDM uptake (AF633+) vs. empty BMDMs (AF633-) was used to quantify total uptake and percentage of BMDM uptake for each strain.

Data for CFU and uptake represent combined data from three experiments (each with 4 or 5 replicates) and were analyzed with Prism using the Kruskal-Wallis test with Dunn’s multiple comparisons.

### *Galleria mellonella* infection

A total of 20 *Galleria mellonella* larvae of cream-white color and similar size were used per strain of *A. fumigatus*. Ten microliters containing 5 × 10^4^ spores was injected directly via Hamilton syringe to the hemocoel as previously described (43). Ten microliters of PBS was administered to the control group to ensure PBS was free from contamination. Additionally, 10 worms that were neither infected nor stabbed were used as a control to check the quality of the worms. After injection, larvae were incubated at 37°C for 5 days. For survival analysis, larvae were monitored three times daily post-injection. The death of larvae was ensured by a lack of movement upon physical stimuli. Log-rank test was performed to determine the survival distribution using GraphPad Prism software. The experiment was done three times with similar results.

### Statistical analyses

Non-parametric (Kruskal-Wallis and log-rank, respectively) tests were used for *ex vivo* assay and virulence assay. Parametric tests (Student’s *t*-test or one-way ANOVA followed by Tukey’s *post hoc* test) were used for other comparisons. GraphPad Prism version 10 was used for all statistical analyses. *P < 0.05, **P < 0.01, ***P < 0.001, ****P < 0.0001.

## SUPPLEMENTAL MATERIAL

Supplemental material msphere.00297-26-s0001.pdf Supplemental figures and tables.

ASM does not own the copyrights to Supplemental Material that may be linked to, or accessed through, an article. The authors have granted ASM a non-exclusive, world-wide license to publish the Supplemental Material files. Please contact the corresponding author directly for reuse.
